# Supplementary material for: Histone H2A variants alpha1-extension helix directs RNF168-mediated ubiquitination
Source: Nat Commun. 2020 May 18;11:2462. doi: 10.1038/s41467-020-16307-4 (PMC7235047; doi:10.1038/s41467-020-16307-4)
Supplement: Supplementary file 1 — Supplementary Information [file 41467_2020_16307_MOESM1_ESM.pdf]

Histone H2A variants alpha1-extension helix directs RNF168-mediated ubiquitination

Jessica, Kelliher et al.

Supplementary information

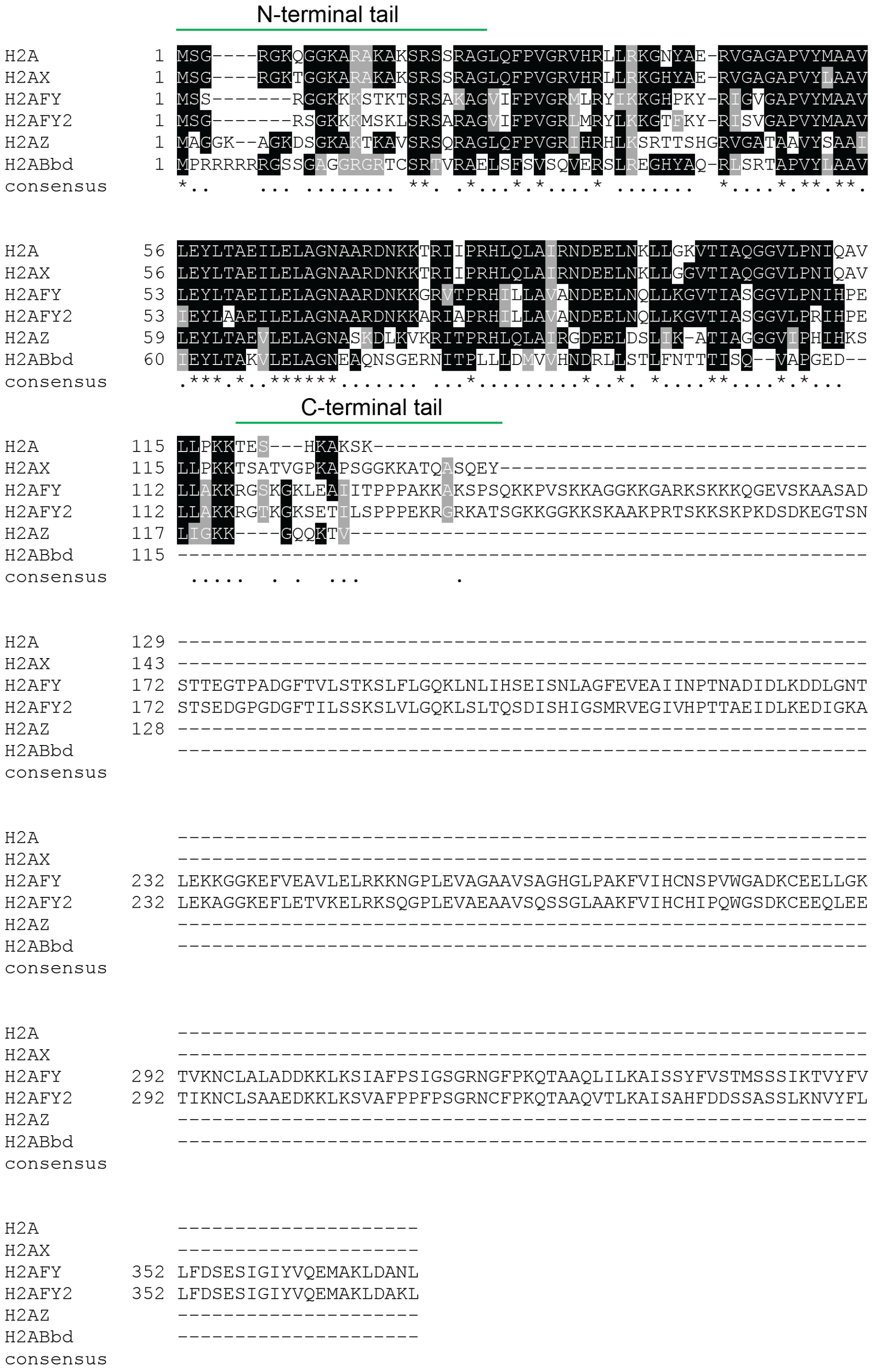

Supplementary Fig. 1. Full sequence alignment of H2A and variants. N-terminal and C-terminal tails were labeled.

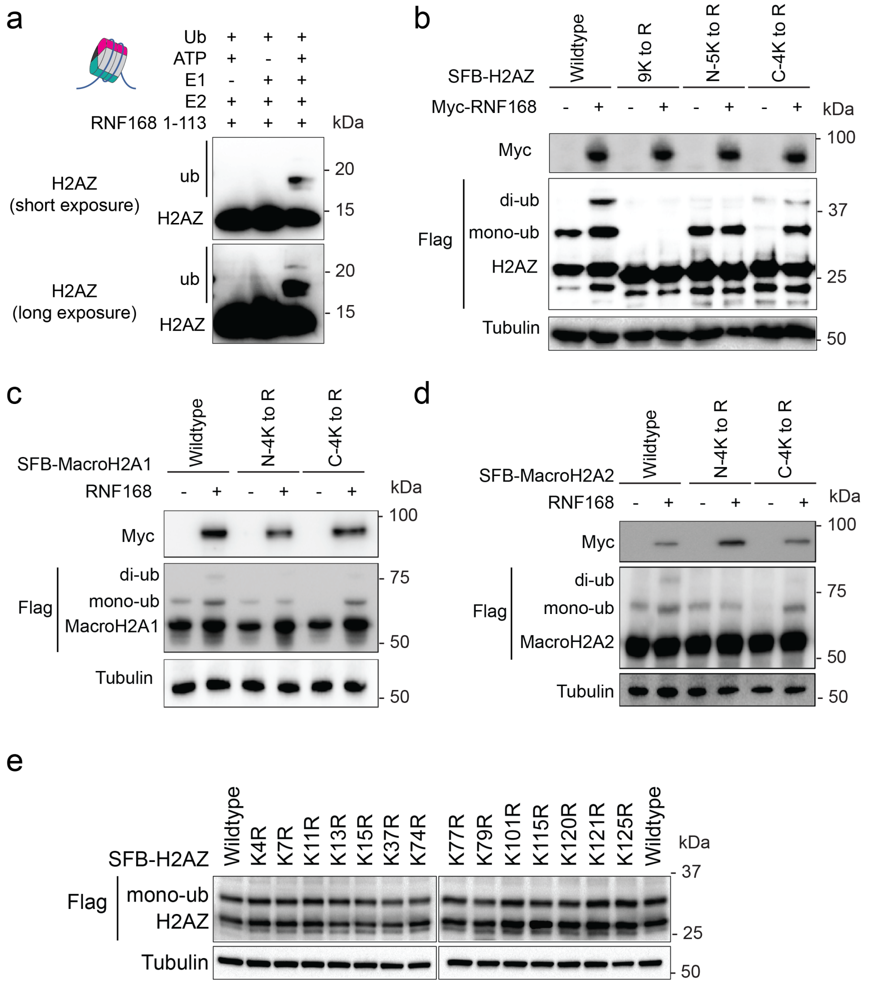

Supplementary Fig. 2. RNF168 ubiquitinates H2A variants. a, in vitro ubiquitination assay of H2AZ-containing nucleosome. H2AZ-containing nucleosome was incubated with ub, ATP, E1, E2 and RNF168 1-113 fragment overnight at 30 ^{o}C. The ubiquitination reaction was stopped by adding SDS-PAGE sample buffer and analyzed by western blot. Repeated two times independently with similar results. **b**. RNF168 ubiquitinates H2AZ at the N-terminal tail. Cells were co-transfected with Myc-RNF168 and SFB-H2AZ mutants as indicated. 9K to R – all nine lysine residues on both histone tails were mutated to arginine; N-5K to R – five N-terminal lysine residues were mutated to arginine; C-4K to R – four C-terminal lysine residues were mutated to arginine. Mono-ubiquitinated H2A variants and di-ubiquitinated H2A variants were labeled as mono-ub and di-ub. Repeated three times with similar results. **c-d**, SFB-MacroH2A1 and MacroH2A2 and their mutants were co-transfected with Myc-RNF168 in HEK293T cells followed by western blot analysis. N-4K to R – N-terminal tail four lysine residues were mutated to arginine, C-4K to R – C-terminal tail four lysine residues were mutated to arginine. Repeated three times with similar results. **e**, SFB-H2AZ KR mutants were transfected in HEK293T cells and analyzed by western blot. Repeated three times with similar results. Source data are provided as Source Data file.

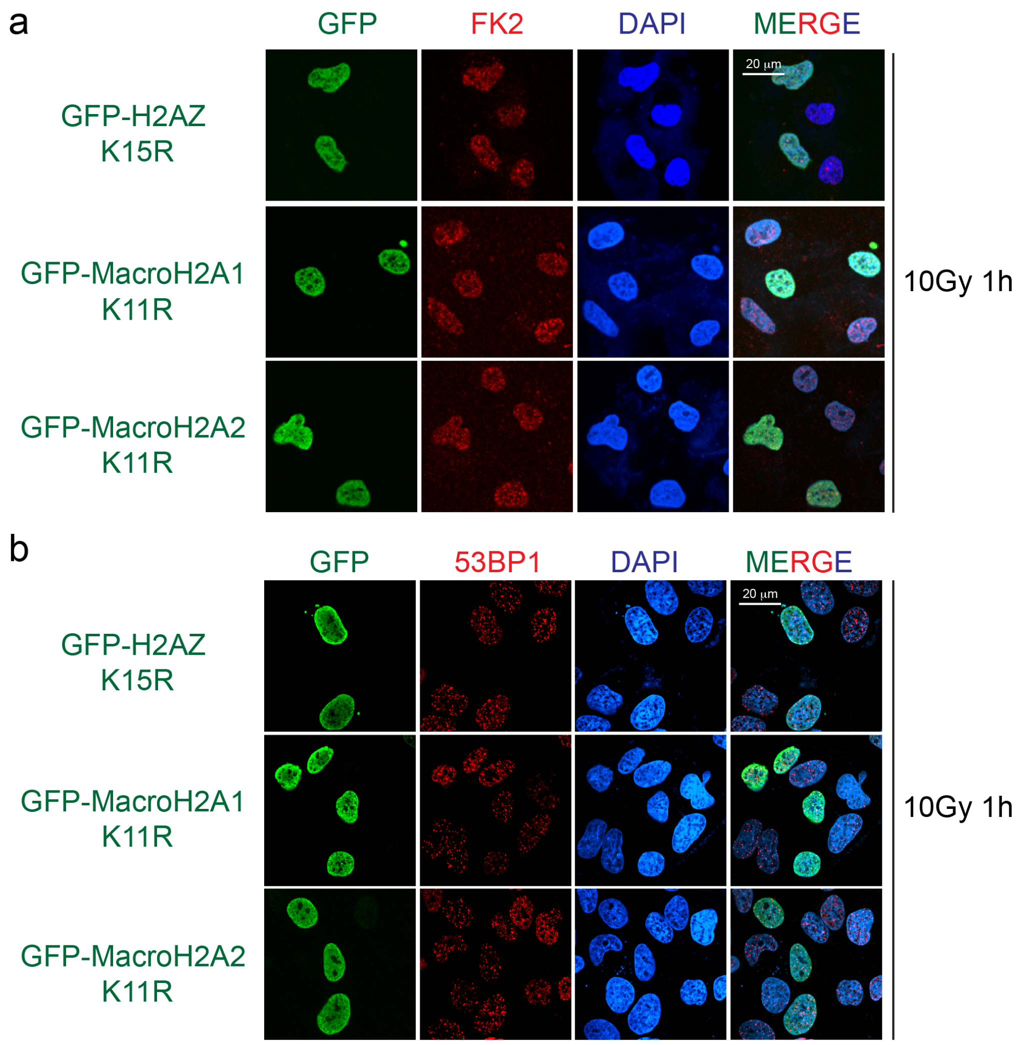

Supplementary Fig. 3. Ectopic expression of H2A variants ubiquitination-defective mutants did not affect DNA damaged ubiquitin signaling and 53BP1 DSB recruitment. a-b, U2OS cells with transient expression of GFP-H2A variants KR mutants were irradiated with 10 Gy and allowed to recover for 1h followed by immunofluorescence analysis using specific antibodies as indicated. Repeated two times independently with similar results.

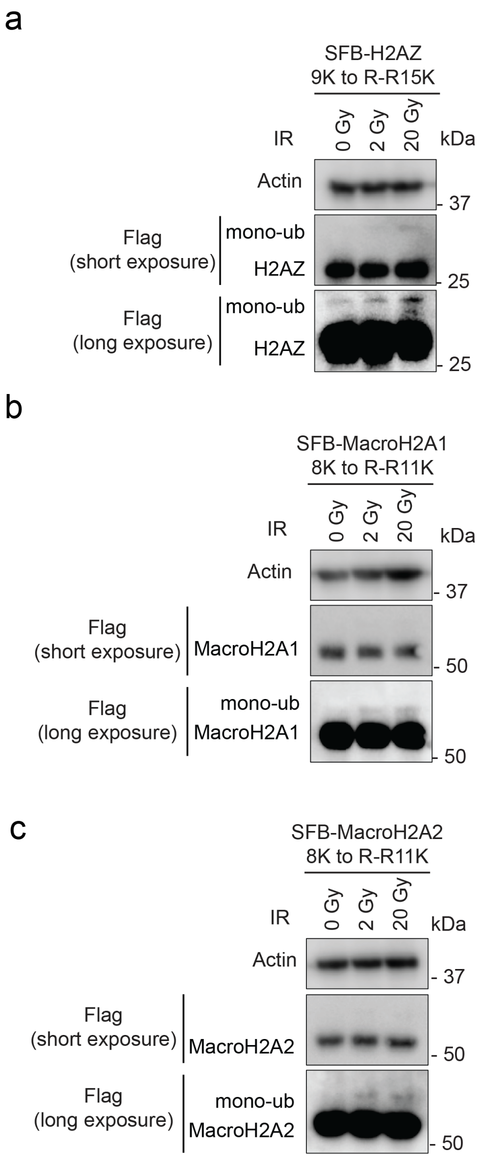

Supplementary Fig. 4. RNF168-targeted H2A variants are DNA damage-induced. a-c, HEK293T stably expressed SFB-H2AZ 9K to R-R15K, SFB-MacroH2A1 8K to R-R11K or SFB-MacroH2A2 8K to R-R11K were irradiated with 2 Gy or 20 Gy. Cells were allowed to recover for 4 h and ubiquitination was analyzed by western blot using antibodies as indicated. Repeated three times independently with similar results. Source data are provided as Source Data file.

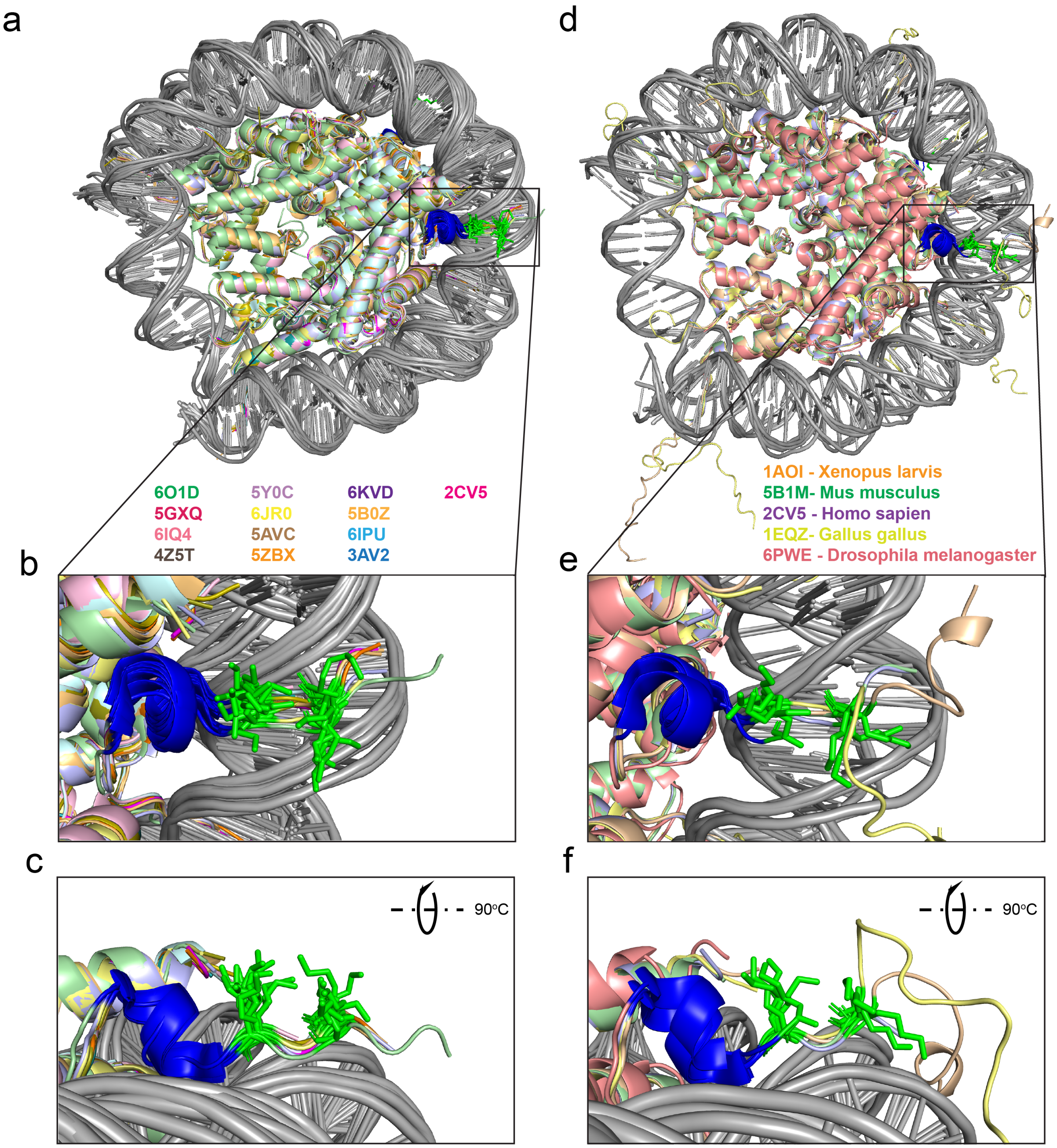

Supplementary Fig. 5. Structural comparison of H2A-containing nucleosomes. **a,** Analysis of the superimposition of thirteen *homo sapiens* H2A-containing nucleosomes. **b-c,** Zoomed illustration of the N-terminal tail region across thirteen *homo sapiens* H2A-containing nucleosomes. **d,** Superimposition of five H2A-containing nucleosomes across different species. **e-f,** Zoomed illustration with two different angles of the N-terminal lysine side chain in five different species. PDB files used in the analysis were listed with corresponding color in the illustration. Alpha-1 extension helix shown in blue. Sidechains of K13 and K15 residues shown in green.

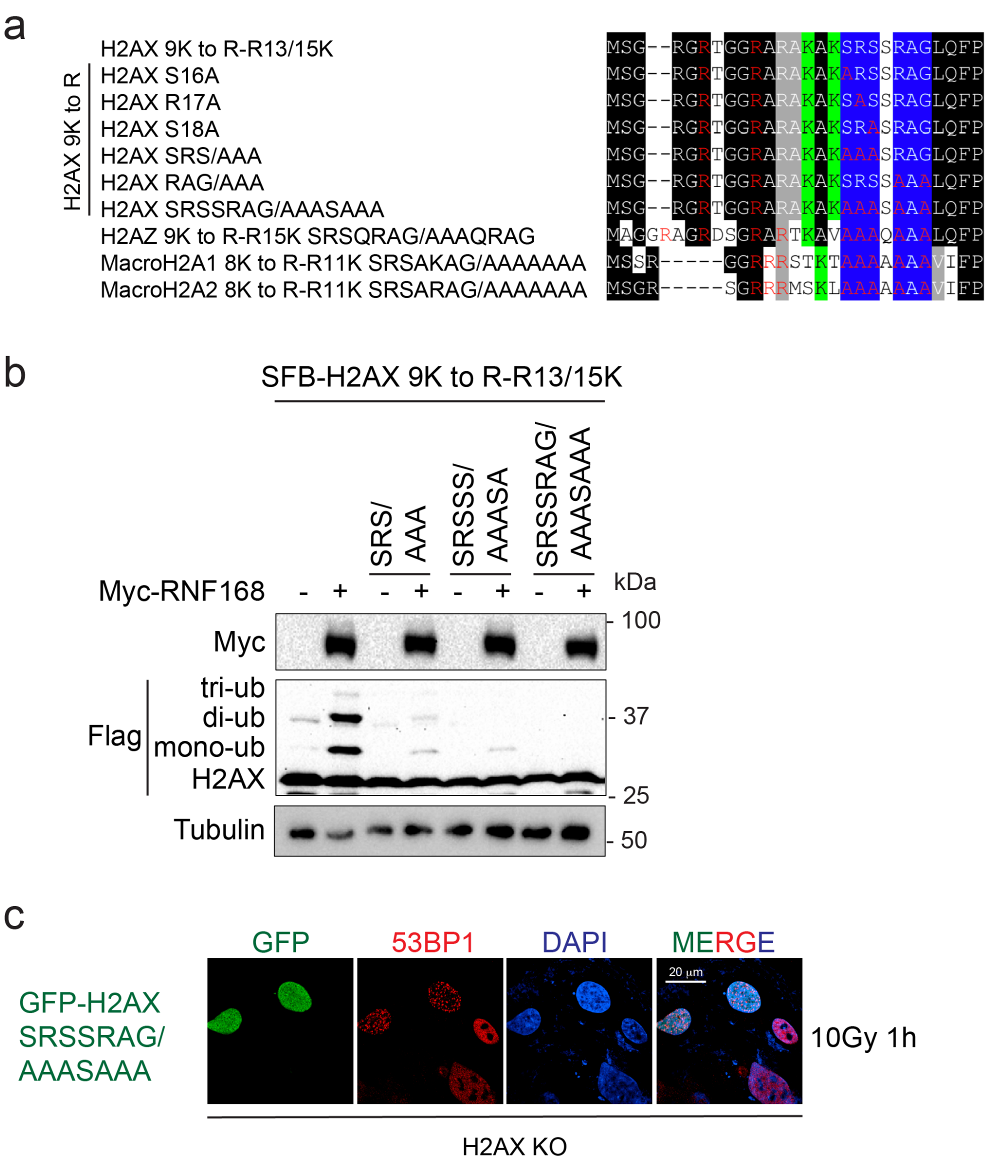

Supplementary Fig. 6. RNF168-targeted site-specific ubiquitination requires the alpha1-extension helix. **a**, Detailed N-terminal sequence illustration of the mutants used in Figure 4 and supplementary figure 4b. **b**, SFB-H2AX mutants were co-transfected with Myc-RNF168 as indicated followed by western blot analysis. c, H2AX alpha1-helix mutation does not affect 53BP1 IRIF formation. Repeated three times independently with similar results. **c**, U2OS H2AX KO cells were transiently transfected with GFP-H2AX SRSSRAG/AAASAAA mutant and irradiated with 10Gy. Cells were then fixed and analyzed by immunofluorescence using 53BP1 specific antibody. Repeated two times independently with similar results. Source data are provided as Source Data file.

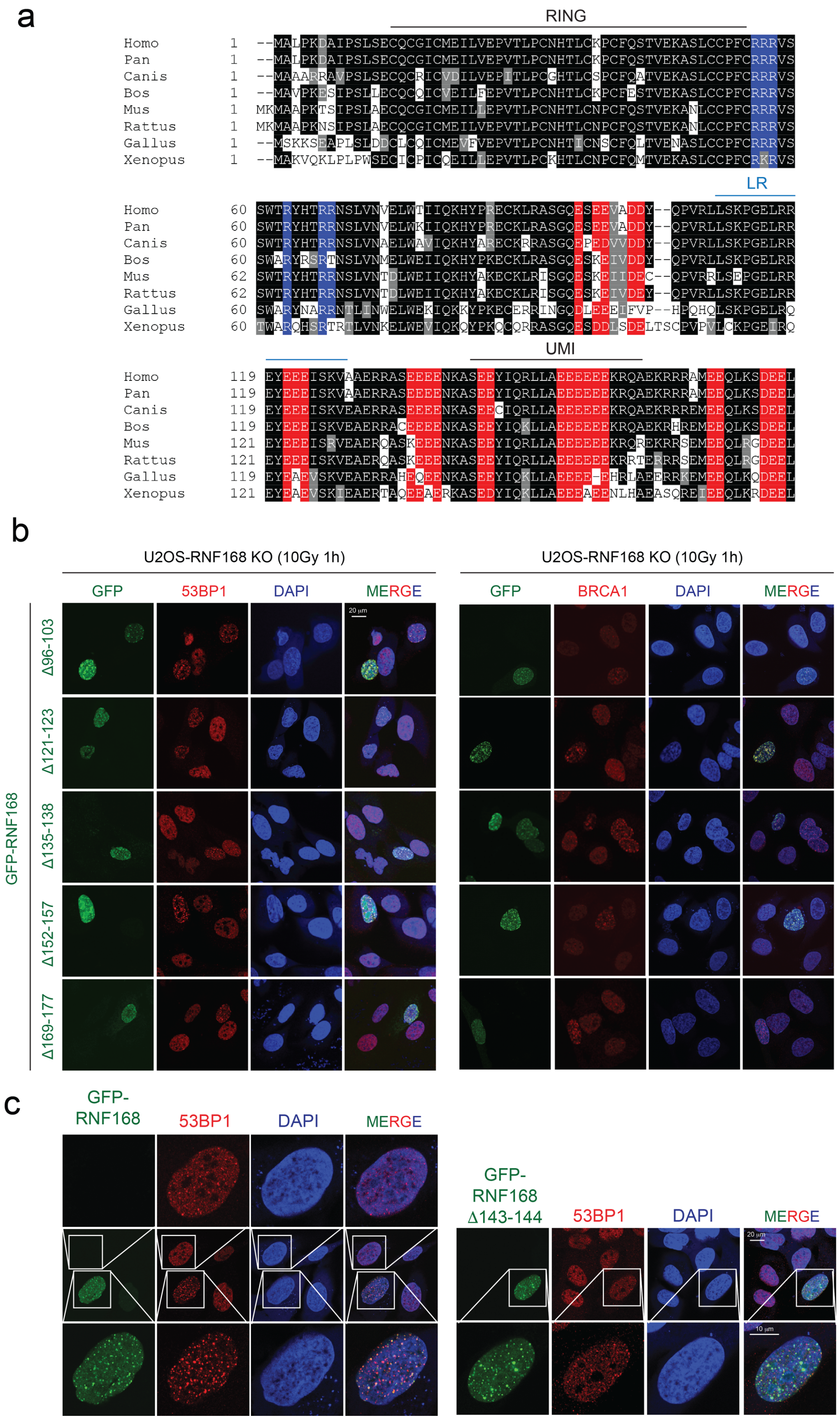
a
RING
LR
UMI
Homo
Pan
Canis
Bos
Mus
Rattus
Gallus
Xenopus
b
U2OS-RNF168 KO (10Gy 1h)
U2OS-RNF168 KO (10Gy 1h)
GFP
53BP1
DAPI
MERGE
GFP
BRCA1
DAPI
MERGE
GFP-RNF168
Δ96-103
Δ121-123
Δ135-138
Δ152-157
Δ169-177
c
GFP-RNF168
53BP1
DAPI
MERGE
GFP-RNF168 Δ143-144
53BP1
DAPI
MERGE

Supplementary Fig. 7. RNF168-acidic region mutants and their effects in DNA repair proteins recruitment to DSBs. **a**, Sequence alignment analysis of RNF168 RING-LRM-UMI domains across species. The arginine anchor (highlighted in blue) within the basic helix following the RING domain. The evolutionary conserved acidic clusters were highlighted in red. **b-c**, GFP-RNF168 mutants were transiently transfected into U2OS RNF168 KO cells. Cells were subjected to 10 Gy and allowed to recover for 1 h. Cells were then fixed and analyzed by immunofluorescence using indicated antibodies. Cells were counterstained with DAPI. Repeated three times independently with similar results.
