# Supplementary material for: Histone H2A variants alpha1-extension helix directs RNF168-mediated ubiquitination
Source: Nat Commun. 2020 May 18;11:2462. doi: 10.1038/s41467-020-16307-4 (PMC7235047; doi:10.1038/s41467-020-16307-4)
Supplement: Supplementary file 3 — Reporting Summary [file 41467_2020_16307_MOESM3_ESM.pdf]

nature research

Corresponding author(s): Justin WC Leung

Last updated by author(s): March 30, 2020

Reporting Summary

Nature Research wishes to improve the reproducibility of the work that we publish. This form provides structure for consistency and transparency in reporting. For further information on Nature Research policies, seeAuthors & Referees and theEditorial Policy Checklist .

Statistics

For all statistical analyses, confirm that the following items are present in the figure legend, table legend, main text, or Methods section.

n/a	Confirmed	
[x]	[ ]	The exact sample size (*n*) for each experimental group/condition, given as a discrete number and unit of measurement
[ ]	[x]	A statement on whether measurements were taken from distinct samples or whether the same sample was measured repeatedly
[ ]	[x]	The statistical test(s) used AND whether they are one- or two-sided<br>*Only common tests should be described solely by name; describe more complex techniques in the Methods section.*
[x]	[ ]	A description of all covariates tested
[ ]	[x]	A description of any assumptions or corrections, such as tests of normality and adjustment for multiple comparisons
[ ]	[x]	A full description of the statistical parameters including central tendency (e.g. means) or other basic estimates (e.g. regression coefficient) AND variation (e.g. standard deviation) or associated estimates of uncertainty (e.g. confidence intervals)
[ ]	[x]	For null hypothesis testing, the test statistic (e.g. *F*, *t*, *r*) with confidence intervals, effect sizes, degrees of freedom and *P* value noted<br>*Give P values as exact values whenever suitable.*
[x]	[ ]	For Bayesian analysis, information on the choice of priors and Markov chain Monte Carlo settings
[x]	[ ]	For hierarchical and complex designs, identification of the appropriate level for tests and full reporting of outcomes
[x]	[ ]	Estimates of effect sizes (e.g. Cohen's *d*, Pearson's *r*), indicating how they were calculated

Our web collection on statistics for biologists contains articles on many of the points above.

Software and code

Policy information about availability of computer code

Data collection	No Software was used for data collection
Data analysis	PyMoL, GraphPad Prism 6, ImageJ 1.51s, Clustal Omega (server), Boxshade 3.21

For manuscripts utilizing custom algorithms or software that are central to the research but not yet described in published literature, software must be made available to editors/reviewers. We strongly encourage code deposition in a community repository (e.g. GitHub). See the Nature Research guidelines for submitting code & software for further information.

Data

Policy information about availability of data

All manuscripts must include a data availability statement. This statement should provide the following information, where applicable:

- Accession codes, unique identifiers, or web links for publicly available datasets
- A list of figures that have associated raw data
- A description of any restrictions on data availability

All data are available from the corresponding author on reasonable request

Field-specific reporting

Please select the one below that is the best fit for your research. If you are not sure, read the appropriate sections before making your selection.

[x] Life sciences [ ] Behavioural & social sciences [ ] Ecological, evolutionary & environmental sciences

For a reference copy of the document with all sections, see nature.com/documents/nr-reporting-summary-flat.pdf

Life sciences study design

All studies must disclose on these points even when the disclosure is negative.

Sample size	Sample sizes were as large as possible given the experimental system used with at least three independent replicates
Data exclusions	none
Replication	All experiments were carried out independently for at least three times
Randomization	Randomization was not necessary, the appropriate controls were used
Blinding	Data collection and Analysis were not performed blindly, we have proper internal controls for every single experiment.

Reporting for specific materials, systems and methods

We require information from authors about some types of materials, experimental systems and methods used in many studies. Here, indicate whether each material, system or method listed is relevant to your study. If you are not sure if a list item applies to your research, read the appropriate section before selecting a response.

Materials & experimental systems

n/a	Involved in the study	
☐	☒	Antibodies
☐	☒	Eukaryotic cell lines
☒	☐	Palaeontology
☒	☐	Animals and other organisms
☒	☐	Human research participants
☒	☐	Clinical data

Methods

n/a	Involved in the study	
☒	☐	ChIP-seq
☒	☐	Flow cytometry
☒	☐	MRI-based neuroimaging

Antibodies

Antibodies used

Flag M2 (Sigma, F1804),
Myc (Santa Cruz, sc-40),
GFP (Invitrogen, A11122),
53BP1 (Novus Biologicals, NB100-304),
BRCA1 (Santa Crus Biotechnology, SC-6954),
γH2AX (Millipore, 05-636),
tubulin (Abcam, AB 6046),
HRP-linked anti-rabbit IgG (Cell signaling, 0704)
HRP-linked anti-mouse IgG (Cell signaling, 0706)
Alexa Fluor 488 goat anti-rabbit (Invitrogen, A11034)
Alexia Fluor 594 goat antimouse (Invirtogen, A11037)
FK2 (Millipore, 04-263)
Actin (Santa Cruz, sc-1616)
H2AZ (Cell Signaling, 2718)
MacroH2A1 (Abcam, ab37264)
MacroH2A2 (Sigma Aldrich, HPA035865)

Validation

Flag M2 (Sigma, F1804), validation reference PMID: 28242625
Myc (Santa Cruz, sc-40), validation reference PMID: 28242625
GFP (Invitrogen, A11122), validation based on manufacturer’s data sheet by wester blotting, validation reference PMID: 29718323
53BP1 (Novus Biologicals, NB100-304), validation reference PMID: 28242625
BRCA1 (Santa Crus Biotechnology, SC-6954), validation reference PMID: 28242625
γH2AX (Millipore, 05-636), validation reference PMID: 28242625
tubulin (Abcam, AB 6046),validation reference PMID: 28242625
HRP-linked anti-rabbit IgG (Cell signaling, 0704) validation based on manufacturer’s data sheet
HRP-linked anti-mouse IgG (Cell signaling, 0706) validation based on manufacturer’s data sheet
Alexa Fluor 488 goat anti-rabbit (Invitrogen, A11034) validation based on manufacturer’s data sheet
Alexia Fluor 594 goat antimouse (Invirtogen, A11037) validation based on manufacturer’s data sheet
FK2 (Millipore, 04-263) validation reference PMID:23812044

Actin (Santa Cruz, sc-1616) validation reference PMID: 25981615
H2AZ (Cell Signaling, 2718) validation reference PMID: 28242625
MacroH2A1 (Abcam, ab37264) validation reference PMID: 28242625
MacroH2A2 (Sigma Aldrich, HPA035865 validation based on manufacturer's data sheet and The Human Protein Atlas project
All Prestige Antibodies Powered by Atlas Antibodies are developed and validated by the Human Protein Atlas (HPA) project (www.proteinatlas.org)and as a result, are supported by the most extensive characterization in the industry. Every Prestige Antibody is tested in the following ways:
• IHC tissue array of 44 normal human tissues and 20 of the most common cancer type tissues.
• Protein array of 364 human recombinant protein fragments.
• Validated for multiple commonly used applications such as IHC (Immunohistochemistry), IF (Immunofluorescence), and WB (Western Blot)

Eukaryotic cell lines

Policy information about cell lines

Cell line source(s)	ATCC: HEK293T (CRL-3216), U2OS (HTB-96)
Authentication	None of the cell lines used were authenticated
Mycoplasma contamination	All cell lines were tested negative for mycoplasma contamination
Commonly misidentified lines (See ICLAC register)	No commonly misidentified cell lines were used
